# Reduced Cortical Complexity in Children with Prader-Willi Syndrome and Its Association with Cognitive Impairment and Developmental Delay

**DOI:** 10.1371/journal.pone.0107320

**Published:** 2014-09-16

**Authors:** Akvile Lukoshe, Anita C. Hokken-Koelega, Aad van der Lugt, Tonya White

**Affiliations:** 1 Dutch Growth Research Foundation, Rotterdam, The Netherlands; 2 Department of Pediatrics, Erasmus Medical Centre Rotterdam/Sophia Children's Hospital Rotterdam Rotterdam, The Netherlands; 3 Department of Child and Adolescent Psychiatry, Erasmus Medical Centre Rotterdam – Sophia Children's Hospital, Rotterdam, The Netherlands; 4 Department of Radiology, Erasmus Medical Centre Rotterdam, Rotterdam, The Netherlands; Vanderbilt University, United States of America

## Abstract

**Background:**

Prader-Willi Syndrome (PWS) is a complex neurogenetic disorder with symptoms involving not only hypothalamic, but also a global, central nervous system dysfunction. Previously, qualitative studies reported polymicrogyria in adults with PWS. However, there have been no quantitative neuroimaging studies of cortical morphology in PWS and no studies to date in children with PWS. Thus, our aim was to investigate and quantify cortical complexity in children with PWS compared to healthy controls. In addition, we investigated differences between genetic subtypes of PWS and the relationship between cortical complexity and intelligence within the PWS group.

**Methods:**

High-resolution structural magnetic resonance images were acquired in 24 children with genetically confirmed PWS (12 carrying a deletion (DEL), 12 with maternal uniparental disomy (mUPD)) and 11 age- and sex-matched typically developing siblings as healthy controls. Local gyrification index (lGI) was obtained using the FreeSurfer software suite.

**Results:**

Four large clusters, two in each hemisphere, comprising frontal, parietal and temporal lobes, had lower lGI in children with PWS, compared to healthy controls. Clusters with lower lGI also had significantly lower cortical surface area in children with PWS. No differences in cortical thickness of the clusters were found between the PWS and healthy controls. lGI correlated significantly with cortical surface area, but not with cortical thickness. Within the PWS group, lGI in both hemispheres correlated with Total IQ and Verbal IQ, but not with Performance IQ. Children with mUPD, compared to children with DEL, had two small clusters with lower lGI in the right hemisphere. lGI of these clusters correlated with cortical surface area, but not with cortical thickness or IQ.

**Conclusions:**

These results suggest that lower cortical complexity in children with PWS partially underlies cognitive impairment and developmental delay, probably due to alterations in gene networks that play a prominent role in early brain development.

## Introduction

Prader-Willi Syndrome (PWS) is a rare and poorly understood neurodevelopmental disorder that affects 1 in 15.000 live births. PWS is caused by the loss of function of paternally inherited genes on the long arm of chromosome 15q11-q13 due to a deletion (DEL, [1]), maternal uniparental disomy (mUPD [2]), unbalanced translocation, or imprinting center defects [3]). PWS is characterized by pre- and postnatal hypotonia, linear growth arrest, endocrine problems, hyperphagia, temper tantrums, skin picking, dysmorphic facial features, high pain threshold, and developmental delays [4]. In addition, individuals with PWS carry a high risk for developing psychiatric illnesses such as psychoses, obsessive-compulsive disorder (OCD), and autism spectrum disorders (ASD) [5], [6].

Key symptoms (short stature, developmental delay and cognitive deficits) indicate that the central nervous system is adversely affected in PWS. To date, only two quantitative structural MRI studies have been performed in adults with PWS. These studies found smaller grey matter volumes in the frontal, temporal and parietal lobes [7], [8] and smaller white matter volumes in the frontal and temporal cortices, brainstem, and cerebellum [8]. Further, Sylvian fissure polymicrogyria was reported in adult patients with PWS in a qualitative structural MRI study [9], suggesting aberrant development of cortical folding in patients with PWS. In children with PWS, we previously reported divergent cortical properties in children with DEL and mUPD, with smaller cortical surface area in children with DEL, but not in children with mUPD, and increased cortical thickness, larger cortical surface cerebrospinal fluid (CSF), and enlarged lateral ventricles in children with mUPD, but not in those with DEL, which could possibly reflect excessive pruning or early cortical atrophy [10]. Since neuronal migration and the resulting cortical thickness, number of cortical columns, cortical surface area, neuronal growth and establishment of cortico-cortical connections are probable underlying factors contributing to gyrification [11]-[13], it is plausible that aberrant early brain development and neuronal migration in children would lead to deviant cortical folding patterns in children with PWS.

Cortical complexity can be quantified using a number of different techniques, including two [11] and three ([14]–[16] dimensional approaches. One novel 3-D approach is the local gyrification index (lGI) [16]. lGI is the ratio between the total surface area of the brain that includes the buried sulcal regions and the pial surface area over the perimeter of the brain within a defined spherical region of interest [16]. The gyrification index is thought to reflect both intra-cortical organization [11] as well as cortico-cortical connectivity [13]. Higher complexity has been shown to be associated with higher cognitive functioning [11]. However, increased gyrification [17] and polymicrogyria [18] has also been reported in patients with autism and adolescents at high genetic risk for schizophrenia who later developed the disorder [19]. In contrast, low gyrification has been associated with mental retardation [20], dyslexia [21], early-onset schizophrenia [22] and velo-cardio-facial syndrome [23], although in the latter increased gyrification was reported as well [24]. Cortical complexity appears to be related not only to cognitive functioning, but also to psychopathology or high-risk states.

In the current study, we explored the cortical complexity in children with PWS by employing the lGI. We hypothesized that children with PWS would show lower cortical complexity, as reported in individuals with mental retardation [20]. Based on our previous data, we expected different gyrification patterns in children with DEL and mUPD, as they also differed in global measures of cortical thickness and cortical surface area [10]. Finally, we investigated whether lower cortical complexity in the PWS group correlated with the Verbal and Performance Intelligence quotient (IQ) of these children, as higher Verbal IQ was reported in children with mUPD compared to those with DEL [25]. Children with mUPD, prior to start of treatment with growth hormone, have lower Performance IQ than those with DEL, but this difference disappears after 4 years of treatment [26].

## Methods

The study population consisted of 29 randomly invited children with PWS who participate in the Dutch Cohort Study [27]. Patients fulfilled the following criteria 1) genetically confirmed PWS; 2) age 6 to 19 years; 3) no history of neurological disorders and 4) no history of psychotropic medication. All children were treated with biosynthetic human growth hormone with the dose of 1 mg/m^2^/day at the time of the study.

Eleven age- and sex-matched, typically developing siblings were included as a control group, fulfilling the following inclusion criteria: 1) age 6 to 19 years; 2) no history of neurological disorders and 3) no history of psychotropic medication.

This study was approved of the Medical Ethical Committee of the Erasmus University Medical Centre in Rotterdam, the Netherlands. Written informed consent was obtained in all cases from the caregivers and children older than 12 years of age. Informed assent was obtained in children younger than 12 years of age.

### Intelligence assessment

For children younger than 7 years, a short form of four subtests (Vocabulary and Similarities (verbal IQ subtests), Block design, and Picture arrangement (performance IQ subtests)) of the Wechsler Preschool and Primary Scale of Intelligence-Revised, Dutch version (WPPSI-R) was used. For children over 7 years, a short form of four subtests (Vocabulary, Similarities (verbal IQ subtests), Block design, and Picture arrangement (performance IQ subtests)) of the Wechsler Intelligence Scale for Children-Revised, Dutch version (WISC-R) was used [28]. Total IQ score was calculated according to an equation based on a normative Dutch outpatient population reference (total IQ = 45.3+2.91×vocabulary standard score + 2.50×block design standard score), combining both WISC-R and WPPSI-R scores [29].

### MRI acquisition

Prior to the MRI scan, all children successfully completed the mock scanner protocol [30]. One of the caregivers was allowed to stay in the MRI suite, close to the child. Imaging was performed on a 3T MRI scanner (750 Discovery, General Electric, Milwaukee, USA), using a dedicated eight-channel head coil. Head motion was minimized by soft cushions between the headphones and head coil and children could view a cartoon during the structural MRI session. Following a 3-plane localizing and coil intensity calibration scans, a high-resolution T1-weighted inversion recovery fast spoiled gradient recalled (IR-FSPGR) sequence was obtained with the following parameters: TR = 10.3 ms, TE = 4.2 ms, TI = 350 ms, NEX = 1, flip angle = 16°, readout bandwidth = 20.8 kHz, matrix 256×256, imaging acceleration factor of 2, and an isotropic resolution of 0.9×0.9×0.9 mm^3^ [30]. T1 sequence duration was 5 min and 40 seconds. All MRI images were reviewed by a qualified radiologist (A. V. D. L.) within 2 weeks after the MRI image acquisition. No gross brain abnormalities were identified.

### Data Preprocessing and Segmentation

Five children with PWS were excluded from the MRI analysis due to motion artifacts and/or failure of proper pial and white surface reconstruction by FreeSurfer (see below). This left 24 eligible patients for analysis (12 children with DEL, 12 children with mUPD). All healthy controls had good quality MRI data.

Cortical reconstruction and volumetric segmentation was performed with the FreeSurfer 5.3 image analysis suite (http://surfer.nmr.mgh.harvard.edu/). The technical details of these procedures are described elsewhere [31]-[33]. Briefly, this processing includes motion correction [34], removal of non-brain tissue [32], [35], automated Talairach transformation, segmentation of the subcortical white matter and deep grey matter volumetric structures [33], [35], intensity normalization [36], automated topology correction [37]. Parcellation of the cerebral cortex into units was performed based on gyral and sulcal structure [38]. FreeSurfer morphometric procedures have been demonstrated to show good test-retest reliability across scanner manufacturers and across field strengths [39]. All FreeSurfer outputs were manually reviewed by a trained researcher who was blinded to the patient data (A. L.) and manual edits were performed where needed to improve the white and pial surface reconstruction.

Cortical surface extracted in the native space of the images was used for the creation of an outer surface, which served as a basis for the lGI calculation. Calculations were performed using Matlab R2011b (Mathworks, Natick, MA). A spherical region of interest with a radius of 25 mm was delineated on the outer surface, and its corresponding region of interest on the cortical surface was identified using a matching algorithm [40]. This process was repeatedly iterated with largely overlapping regions of interest, resulting in cortical maps of gyrification for subsequent statistical comparisons [40].

### Statistical analysis

General linear model implemented in QDEC (FreeSurfer, v5.3) was used to investigate differences in lGI (smoothing factor 5 mm) in children with PWS and healthy controls at each vertex of the surface (p<0.05). Left and right hemispheres were tested separately. We accounted for age effects, as it has been shown that lGI decreases with age from early childhood [41]. To correct for vertex-by-vertex multiple comparisons, Monte Carlo cluster simulation with 10,000 iterations and cluster analysis was performed to identify the clusters of significant cortical complexity (p<0.05). All results are reported after Monte Carlo correction, unless otherwise indicated.

The properties of the significant clusters after the correction for multiple comparisons were further explored. The cortical surface area and cortical thickness information of the clusters was exported from QDEC to SPSS (version 20, IBM Corporation, Armonk, NY, USA). Nonparametric Mann-Whitney U-tests were performed to investigate the group differences in cortical surface area/cortical thickness of the clusters.

Spearman's rho correlations were used to estimate the relationships between lGI and cortical surface area/cortical thickness in children with PWS and healthy controls, as well as the relations between lGI and IQ in children with DEL and those with mUPD. Fisher's r-to-z transformations were performed to investigate the differences between the groups (PWS vs. HC or DEL vs. mUPD), and if no differences were found, groups were combined for further analyses.

## Results

Clinical data are presented in Table 1. No significant differences were found in age, handedness, and gender distribution among groups. Furthermore, there were no differences in IQ or age at the start of growth hormone treatment. Two children in the mUPD group were diagnosed with an autism spectrum disorder. None of the children had a history of treatment with psychotropic medication.

**Table 1:** Demographic data of the participant.

	PWS		Control	p value
	DEL	mUPD		
**Age** (years)	12.6 (3.2)	11.5 (4.0)	11.7 (2.7)	.60
**Age range (years)**	6.7–17.0	6.1–18.4	7.1–15.8	
**Sample size (N)**	12	12	11	.97
**Gender**				.14
Male	5	4	8	
Female	7	8	3	
**Handedness (N)**				
Left	2	1	1	
Right	10	9	10	
Ambidextrous	0	2	1	
**Age at start of GH treatment**	5.8 (2.9)	4.8 (2.5)		.42
**Total IQ Score**	70.0 (15.8)	70.1 (16.1)		.95
Vocabulary subtest SS	4.4 (3.4)	4.6 (3.8)		1.0
Block design subtest SS	4.8 (2.7)	4.6 (2.6)		.84
**Psychiatric history**	0	2*		
**Use of Psychotropic medication**	0	0		

Data expressed as mean (SD) or number. PWS – Prader-Willi Syndrome; DEL – deletion; mUPD – maternal uniparental disomy; SD – standard deviation; SS – standard score. No significant differences were found in either age or gender distribution across groups.

* Two children diagnosed with Autism Spectrum Disorder prior to MRI scan

### lGI in PWS and healthy controls

**Figure 1:**
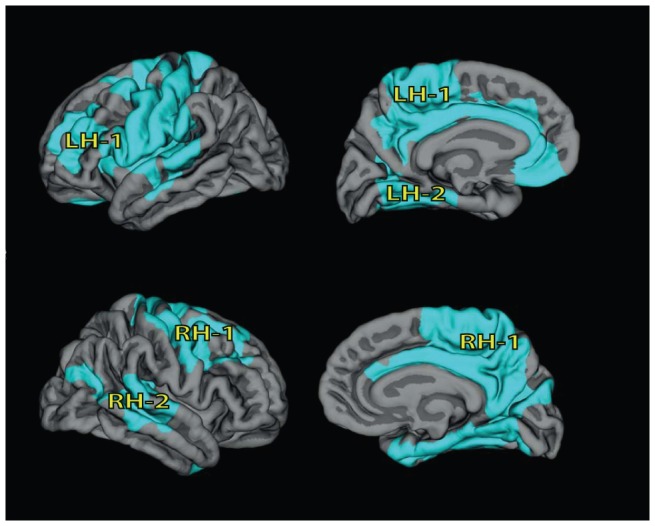
Differences in lGI between children with PWS and healthy controls. Top row: left hemisphere (LH). Bottom row: right hemisphere (RH). We found lower lGI in children with PWS, compared to healthy controls (areas indicated in light blue, p<0001, after correction for multiple testing). Results are corrected for age.

Differences in lGI between children with PWS and healthy controls are presented in Figure 1 and Table 2. In the left hemisphere, two clusters (LH-1 and LH-2) were found to have significantly lower lGI in children with PWS. LH-1 (p<.0001) included a relatively large cortical area, comprising precentral, postcentral and paracentral gyri, superior frontal gyrus, caudal middle frontal, superior parietal, supramarginal gyrus, medial and lateral orbitofrontal gyri, precuneus, anterior and posterior cingulate gyri (Table 2). LH-2 (p<.001) included rostral middle frontal gyrus, pars opercularis, pars triangularis, insula, superior, middle and transverse temporal gyri. In the right hemisphere, two clusters (RH-1 and RH-2) were identified with significantly lower gyrification in children with PWS. RH-1 (p<.0001) comprised the precentral, postcentral and paracentral gyri, superior frontal gyrus, middle frontal gyrus, precuneus, caudal anterior, posterior and isthmus cingulate gyri, lingual gyrus, fusiform gyrus, parahippocampal gyrus and entorhinal gyrus (Table 2). RH-2 (p<.001) included inferior parietal gyrus, middle and superior temporal gyri, and the banks of superior temporal sulcus (Table 2).

**Table 2:** Clusters with lower gyrification in children with PWS compared to healthy controls.

lGI Cluster	Cluster includes	Talairach coordinates (x, y, z)	Cluster size (mm^2^)	p value*
LH-1	Precentral, postcentral, paracentral, superior frontal, superior parietal, caudal middle frontal, rostral middle frontal, supramarginal, medial and lateral orbitofrontal, precuneus, anterior and posterior cingulate, isthmus cingulate, pars triangularis, pars opercularis, insula, superior, middle and transverse temporal	(−36.8, −18.3, 64.5)	28169.3	<.0001
LH-2	Pericalcarine, parahippocampal, lingual, fusiform	(−21.4, −61.3, 8.9)	3515.7	<.001
RH-1	Precentral, postcentral, paracentral, supramarginal, superior frontal, rostral and caudal middle frontal, precuneus, cuneus, caudal anterior and posterior cingulate, isthmus cingulate, lingual, fusiform, parahippocampal, entorhinal, pericalcarine	(9.4, −50.8, 47.8)	22246.5	<.0001
RH-2	Inferior parietal, middle and superior temporal, banks of superior temporal sulcus	(45.9, −43.8, 7.5)	4669.8	<.0001

Clusters are also presented in Figure 1. LH – Left hemisphere; RH – Right Hemisphere.

*p value for comparison between children with PWS and healthy controls

### Cortical surface area and cortical thickness in PWS and healthy controls

Smaller cortical surface area was found in children with PWS, compared to healthy controls in all four lGI clusters (left: LH-1: p = .006, LH-2: p = .001; right: RH-1: p = .004, RH-2: p = .008) (Table 3).

**Table 3:** Top part: Mean cortical surface area, cortical thickness in children with PWS and healthy controls in clusters with low lGI. Bottom part: correlations between lGI and cortical surface area/cortical thickness within the clusters in each group and the differences between the groups in their relationships of lGI and cortical surface area/thickness.

	Cortical Surface Area				Cortical Thickness			
	PWS	Control		Correlation with lGI^1^	PWS	Control		Correlation with lGI^1^
lGI cluster	Mean (SD)	Mean (SD)	p value	rho (p value)	Mean (SD)	Mean (SD)	p value	rho (p value)
LH-1	35567 (3828)	40139 (4064)	**p<.01**	**.75 (p<.0001)**	2.81 (.173)	2.77 (.099)	.26	−.07 (ns)
LH-2	4171 (518)	4914 (437)	**p<.01**	**.77 (p<.0001)**	2.44 (.207)	2.57 (.136)	.06	.25 (ns)
RH-1	27724 (2947)	31278 (2662)	**p<.01**	**.86 (p<.0001)**	2.62 (.136)	2.64 (.129)	.49	−.08 (ns)
RH-2	6065 (724)	7006 (935)	**p<.01**	**.72 (p<.0001)**	3.00 (.181)	2.92 (.139)	.22	**−.38 (p<.05)**

	Cortical Surface Area			Cortical Thickness			
	PWS	Control	Between groups Fisher's r-to-z	PWS	Control		Between groups Fisher's r-to-z
	rho (p value)	rho (p value)	z (p value)	rho (p value)	rho (p value)		z (p value)
LH-1	**.64 (p<.01)**	**.88 (p<.0001)**	−1.49 (ns)	−.06 (ns)	.26 (ns)		−.79 (ns)
LH-2	**.67 (p<.0001)**	**.76 (p<.01)**	−.45 (ns)	.06 (ns)	.30 (ns)		−.60 (ns)
RH-1	**.82 (p<.0001)**	**.74 (p<.05)**	.50 (ns)	−.21 (ns)	.03 (ns)		−.06 (ns)
RH-2	**.58 (p<.01)**	**.**60 (p = .051)	−.07 (ns)	−.39 (ns)	−.12 (ns)		−.70 (ns)

Top: Cortical surface area was significantly lower in children with PWS than in healthy controls and no differences were found in cortical thickness. lGI correlated with cortical surface area, but not with cortical thickness in the combined sample. Bottom: lGI correlated with cortical surface area, but not cortical thickness in PWS and healthy controls. No differences between the groups were found in their relationship between lGI and cortical surface area/cortical thickness. LH – Left hemisphere; RH – Right Hemisphere; SD – standard deviation, rho –Spearman's rho.

1 Correlations were calculated in total group (PWS + HC)

No differences in cortical thickness between children with PWS and healthy controls were found within the clusters (Table 3).

### Relation between lGI and cortical surface area as well as cortical thickness

No differences were found regarding groups' relationships between lGI and cortical surface area (Fisher's r-to-z: LH-1: z = −1.49, ns; LH-2: z = −.45, ns; RH-1: z = .50, ns; RH-2: z = −.07, ns), and cortical thickness (Fisher's r-to-z: LH-1: z = −.79, ns; LH-2: z = −.60, ns; RH-1: z = −.06, ns; RH-2: rho = −.70, ns) therefore PWS and healthy control groups were combined for further analyses (Table 3).

lGI correlated positively with cortical surface area within these clusters (LH-1: rho = .75, p<.0001; LH-2: rho = .77, p<.001; RH-1: rho = .86, p<.0001; RH-2: rho = .72, p<.0001).

lGI negatively correlated with cortical thickness within only one cluster in the right hemisphere (LH-1: rho = −.07, ns; LH-2: rho = .25, ns; RH-1: rho = −.08, ns; RH-2: rho = −.38 (p<.05)

### Relation between lGI and IQ measures in children with PWS

No differences were found between DEL and mUPD relation between lGI and Total, Verbal and performance IQ, therefore subtypes were combined for further analyses (Table S1).

Correlations between lGI and total, performance and verbal IQ in children with PWS are reported in Table 4. In the left hemisphere, lGI of LH-1, comprising mainly frontal areas, was positively correlated with total IQ (p<.05), verbal IQ (p<.01), but not with performance IQ (ns). lGI of LH-2 was positively correlated with verbal IQ only (p<.05). In the right hemisphere, lGI of RH-1, comprising mainly frontal areas, was positively associated with verbal IQ (p<.05), but not with total IQ or performance IQ. No relation was found between lGI of RH-2 and IQ scores.

**Table 4:** Correlations between gyrification index in left and right hemispheres and IQ in children with PWS.

lGI cluster	Total IQ		Verbal IQ		Performance IQ	
	rho	p value	rho	p value	rho	p value
**LH-1**	**.44**	**.033**	**.50**	**.013**	.30	.15
**LH-2**	.36	.08	**.47**	**.022**	.20	.36
**RH-1**	.36	.08	**.42**	**.044**	.23	.28
**RH-2**	.07	.76	.11	.63	.05	.82

LH – Left hemisphere; RH – Right Hemisphere; SD – standard deviation, rho –Spearman's rho.

We further explored which brain areas within the LH-1 and RH-1 clusters showed an association between lGI and IQ (Table S2 and Table S3, for left and right hemisphere, respectively). Within the LH-1, a strong correlation with total IQ was found in precentral and postcentral gyri (p<.01 and p<.05, respectively), superior parietal gyrus (p<.01), caudal middle frontal gyrus (p<.01), rostral middle frontal (p<.05), superior frontal (p<.01), precuneus (p<.05) and pars triangularis (p<.05). Verbal IQ was strongly associated with precentral (p<.01), postcentral (p<.01) and paracentral (p<.05) gyri, superior parietal gyrus (p<.01), caudal middle frontal gyrus (p<.01), rostral middle frontal (p<.01), superior frontal (p<.01), precuneus (p<.01), pars opercularis (p<.05), and pars triangularis (p<.05). Performance IQ was associated with the lGI of precentral gyrus (p<.05), only.

Within the LH-2, lGI of fusiform gyrus (p<.05) and pericalcarine (p<.05) positively correlated with performance IQ. No associations were found with Total or Verbal IQ.

In the right hemisphere (Table S3), a positive association between lGI and total IQ was found in precentral (p<.01), postcentral (p<.01), and paracentral (p<.05) gyri, caudal middle frontal (p<.01) and superior frontal gyri (p<.05). Verbal IQ was positively associated with lGI in precentral (p<.01), postcentral (p<.05) and paracentral gyri (p<.05), caudal middle frontal gyrus (p<.001) and superior frontal (p<.05). Performance IQ was associated with lGI in precentral gyrus (p<.05), and caudal middle frontal gyrus (p<.05).

In the right hemisphere, within the RH-2, lGI of inferior parietal gyrus correlated positively with Total IQ (p<.05) and verbal IQ (p<.05).

### Differences in lGI between children with DEL and mUPD

No differences in lGI between children with DEL and mUPD were found in the left hemisphere. In the right hemisphere, two clusters were found with significantly smaller lGI values in children with mUPD compared to those with DEL (Table 5). Cluster RH-3 (p<.001) comprised right precentral gyrus, pars triangularis, pars opercularis and insula. RH-4 (p<.01) comprised pericalcarine, cuneus, lingual, and lateral occipital gyri.

**Table 5:** Clusters with lower gyrification in children with mUPD compared to children with DEL.

lGI Cluster	Cluster includes	Talairach coordinates (x, y, z)	Cluster size (mm^2^)	p value*
RH-3	Precentral, pars opercularis, pars triangularis, insula	(51.0, 29.9, 3.3)	1925	<.001
RH-4	Pericalcarine, cuneus, lingual, lateral occipital	(13.6, −91.5, 15.7)	2212	<.01

RH – Right Hemisphere.

* p value for comparison between children with PWS and healthy controls

The cortical surface area within RH-3 and RH-4 clusters did not significantly differ between children with DEL and those with mUPD (Table 6). Cortical thickness was greater in children with mUPD within RH-3 (p = .05), but not within RH-4, compared to children with DEL.

**Table 6:** Top part: Mean cortical surface area, cortical thickness in children with DEL and mUPD in clusters with low lGI. Bottom part: correlations between lGI and cortical surface area/cortical thickness within the clusters in each subtype and the differences between the subtypes in their relationships of lGI and cortical surface area/thickness.

	Cortical Surface Area					Cortical Thickness				
lGI cluster	DEL	mUPD		Correlation with lGI^1^		DEL	mUPD		Correlation with lGI^1^	
	Mean (SD)	Mean (SD)	p value	rho (p value)		Mean (SD)	Mean (SD)	p value	rho (p value)	
RH-3	2068.8 (202.5)	1990.6 (137.5)	.42	**.47 (p<.05)**		2.91 (.14)	3.05 (.15)	**.05**	−.10 (ns)	
RH-4	1823.4 (126.8)	1709.8 (234.3)	.20	**.66 (p<.0001)**		1.92 (.11)	1.96 (.24)	.95	−.24 (ns)	

	Cortical Surface Area					Cortical Thickness				
	DEL	mUPD		Between groups Fisher's r-to-z		DEL	mUPD		Between groups Fisher's r-to-z	
	rho (p value)	rho (p value)		z (p value)		rho (p value)	rho (p value)		z (p value)	
RH-3	.41 (ns)	.39 (ns)			−.05 (ns)	.27 (ns)	−.06 (ns)		.71(ns)	
RH-4	**.61 (p<.05)**	**.71 (p<.01)**			−.38 (ns)	−.48 (ns)	−.07 (ns)		−.96 (ns)	

Top: There were no differences in cortical surface area between the genetic subtypes. mUPD had significantly larger cortical thickness in RH-1 only. lGI correlated with cortical surface area, but not with cortical thickness in the combined sample. Bottom: lGI correlated with cortical surface area (except in RH-1 in DEL), but not with cortical thickness in both DEL and mUPD. No differences between the subtypes were found in their relationship between lGI and cortical surface area/cortical thickness. LH – Left hemisphere; RH – Right Hemisphere; SD – standard deviation, rho –Spearman's rho.

1 Correlations are calculated in total group (DEL + mUPD).

No differences were found regarding subtypes' relationships between lGI and cortical surface area (Fisher's r-to-z: RH-3: z = −.05, ns; Rh-4: z = −.38, ns), and cortical thickness (Fisher's r-to-z: RH-3: z = .71, ns; RH-4: z = −.96, ns) therefore DEL and mUPD subtypes were combined for further analyses (Table 6).

lGI of both clusters significantly correlated with cortical surface area (RH-3: rho = .47, p<.05; RH-4: rho = .66, p<.0001), but not with cortical thickness (RH-3: rho = −.10, ns; RH-4: rho = −.24, ns).

No significant correlations were observed between lGI of these clusters and Verbal, Performance or Total IQ.

## Discussion

This is the first study investigating cortical complexity in children with PWS. We hypothesized that children with PWS would have lower gyrification compared to healthy controls, as it has been reported in individuals with mental retardation [20]. Furthermore, we expected that within the PWS group, children with DEL and mUPD would show divergent gyrification patterns, as they also differed in global measures of cortical thickness and cortical surface area [10]. We found that children with PWS had lower lGI in mainly the frontal, but also in the temporal and parietal lobes. Cortical areas with low lGI were characterized by lower cortical surface area, but normal cortical thickness in children with PWS. Furthermore, lower gyrification in the frontal lobes correlated with lower IQ in children with PWS. Compared to children with DEL, children with mUPD had lower lGI in two small clusters in the right hemisphere, comprising precentral gyrus, pars triangularis, pars opercularis and insula, cuneus, lingual, and lateral occipital gyri. No differences were found between the subtypes in cortical surface area of these clusters. Children with mUPD had larger cortical thickness in a cluster comprising the right inferior frontal gyrus and insula, in concert with our previous study, were we reported larger global cortical thickness in children with mUPD [10]. Also in these clusters, significant correlation was found between lGI and cortical surface area, but not with cortical thickness.

We found lower cortical gyrification in the fronto-temporo-parietal cortical areas in children with PWS, irrespective of the genetic subtype. Interestingly, most areas with smaller lGI, in combination with smaller cortical surface area, were identified bilaterally, indicating aberrant global developmental processes. Our results are well in concert with other studies in patients with cognitive impairment, showing lower gyrification in patients with non-specific mental retardation [20], [42], and children with velo-cardio-facial (deletion 22q11.2) syndrome [23], [43]. We found a strong correlation between the lGI of these areas and the verbal and total IQ score in the PWS group. Fronto-parietal cortical areas are implicated in higher cognitive function such as working memory and attention regulation [44] (rostral middle frontal gyrus, which includes dorsolateral prefrontal cortex), visuospatial processing, memory and self-awareness (precuneus, superior frontal gyrus) [45], language perception, processing and production (pars opercularis (Broca's area), supramarginal gyrus) [46], somatosensory integration and processing (precentral, postcentral and paracentral gyri) [47]. Although we did not measure IQ in healthy controls, robust positive association between cortical complexity and intelligence has been reported in typically developing children [48] and adults [49].

The evolutionary emergence of higher cognitive functions in human primates may at least be partially driven by the expansion of the prefrontal cortical surface area and an increase in cortical complexity across species [12]. In addition, cortical surface area and cortical complexity are highly correlated in the rostral parts of the brain, especially prefrontal cortex in healthy human individuals [50] – in the current study we report same positive relationship between lGI and cortical surface area in both healthy controls and children with PWS. These results suggest that smaller lGI, together with smaller cortical surface area, might partially underlie the cognitive impairment in children with PWS.

The absence of large differences in cortical gyrification between the genetic subtypes of PWS might partially stem from the absent genetic expression of the PWS region on chromosome 15q11-q13, in both subtypes. Two PWS locus genes have recently been implicated in regulation of neuronal migration and maturation, namely human necdin encoding gene (NDN) and ubiquitin protein ligase E3A (UBE3A). NDN is found to be involved in the tangential migration of the GABAergic interneurons during early fetal development [51] and migration of sympathetic neurons [52]. NDN is known to have proliferation promoting properties, however, knockout of necdin resulted in no differences in cortical thickness in mice [53], suggesting that proliferation-promoting characteristics were counteracted by other genetic factors [53]. UBE3A was recently reported to be paternally expressed by immature neurons in prenatal brain development [54]; once matured, these neurons expressed the maternal UBE3A allele [54]. This implicates that all individuals with PWS with either DEL or mUPD lack paternal UBE3A expression before birth. After birth, UBE3A is expressed unaltered in individuals with DEL and is overexpressed in those with mUPD [55]. It is to date unknown whether paternal UBE3A is required for a neuron to mature or whether it is a neuronal-maturation-dependent regulator of maternal UBE3A expression and postnatal functioning of the mature neurons [54]. Further, UBE3A-expressing neurons are mostly superficial neurons, innervating layers IV-VI of the cortex [54]. Both UBE3A knockout as well as overexpression results in aberrant dendritic tree formation [56]. Given that cortex develops in an inside-out fashion with early-born neurons occupying deeper layers, the absence of paternal UBE3A gene will affect mainly late-born neurons and thus outer layers of the cortex, while the absence of paternal NDN will have an impact on interneurons throughout cortical layers [51]. Given that NDN regulates migration of interneurons, and UBE3A is involved in the maturation of late-born neurons, it is therefore plausible that lack of these two genes would result in brain-wide deficits of the cortical mantle formation due to effects on intra-cortical connectivity, however having limited effect on cortical thickness in children with PWS.

Converging evidence suggests that PWS genetic locus plays an important role in other neurodevelopmental disorders, such as Rett [57], Fragile × [58] syndromes and autism [57]. MECP2, loss of which causes Rett syndrome, regulates the expression of 15q11.2-13 genes by binding on imprinting centre and modifying chromatin condensation. FMR1 directly interacts with CYFIP1 in controlling neural connectivity [59]. Expression of UBE3A gene was reduced in Angelman, Rett and autism post mortem brain samples [57]. All in all these studies suggest that PWS locus genes take part in a large genetic network, which regulates neurodevelopment and likely underlies intellectual disability and developmental delay in these disorders.

Early post-mortem studies of the gyrification index suggests that it is stable over the lifetime [11]. However, MRI studies suggest that gyrification patterns change during childhood and adolescence [41], which is likely to be influenced by optimization of neuronal connections and pruning and may reflect the emergence of higher cognitive functions [13], [41], [60]. Interestingly, we found primarily lower cortical complexity in the frontal and temporal lobes in children with PWS. In typically developing children, frontal lobes are found to mature later during cerebral development and only after phylogenetically older cortical areas have matured [61]. The older cortical areas include inferior medial frontal and temporal lobes [61], which we found to have a lower lGI in PWS. The ontologically newer cortical areas include the superior parietal and prefrontal cortices, which we found to have significantly lower lGI values in PWS. Based on these data, our findings suggest that incomplete maturation or developmental delay of the evolutionarily older cortices might have contributed to the aberrant gyrification of the frontal cortex, which might underlie the deficit in higher cognitive function seen in patients with PWS.

Our results are limited by the small sample size, thus generalization to broader PWS population should be done with great caution. Further, our control group consists of age and gender matched healthy siblings. A possible concern of recruiting siblings as control group is that volumes of most brain structures are heritable [62]. However, as PWS occurs due to a *de novo* genetic event during conception, we assume that unaffected siblings are representative of a random sample of the general population. The great advantage of having siblings as control group is that the effects of possible environmental and hereditary factors on brain development are greatly reduced, and that the observed significant differences are more likely PWS-specific.

## Conclusions

In conclusion, we found that children with PWS show lower cortical complexity in frontal, temporal and parietal lobes when compared to healthy controls, irrespective of their specific genotype. These results suggest that lower cortical complexity in children with PWS partially underlies cognitive impairment and developmental delay.

## Supporting Information

Figure S1 **Correlations between lGI, cortical surface area and cortical thickness in children with PWS and healthy controls.** LH – Left hemisphere; RH – Right hemisphere. lGI correlated with cortical surface area in PWS and healthy controls, but not with cortical thickness. For corresponding Spearman's rho and p values, together with Fisher's r-to-z transformations for group differences in their relation between lGI and cortical surface area/cortical thickness please see Table 3.
(TIF)

Figure S2 **Correlations between lGI and TIQ in children with DEL and mUPD.** LH – Left Hemisphere; RH – Right Hemisphere. lGI correlated significantly with Total IQ in children with DEL and mUPD, and no differences were found between the genetic subtypes of PWS. For corresponding Spearman's rho and p values, together with Fisher's r-to-z transformations for subtype differences in their relation between lGI and IQ please see Table S1.
(TIF)

Table S1 **lGI and IQ relationship within mUPD and DEL groups and group differences.** LH – Left hemisphere; RH – Right hemisphere, rho –Spearman's rho. No differences were found between the genetic subtype in the relationship of lGI and IQ measures.
(DOCX)

Table S2 **Correlations between lGI and IQ in clusters with lower lGI in the left hemisphere in patients with PWS.** LH – left hemisphere, rho –Spearman's rho. The correlation of lGI and IQ measures (Total, Verbal, Performance) per anatomical area, according to Destrieux anatomical atlas, that clusters comprised of. lGI correlated mainly with Verbal and Total IQ but less with Performance IQ.
(DOCX)

Table S3 **Correlations between lGI and IQ in clusters with lower lGI in the right hemisphere in patients with PWS.** RH – Right hemisphere, rho –Spearman's rho. The correlation of lGI and IQ measures (Total, Verbal, Performance) per anatomical area, according to Destrieux anatomical atlas, that clusters comprised of. lGI correlated mainly with Verbal and Total IQ, but less with Performance IQ.
(DOCX)
