# Supplementary figures and images for: Reduced Cortical Complexity in Children with Prader-Willi Syndrome and Its Association with Cognitive Impairment and Developmental Delay
Source: PLoS One. 2014 Sep 16;9(9):e107320. doi: 10.1371/journal.pone.0107320 (PMC4165760; doi:10.1371/journal.pone.0107320)

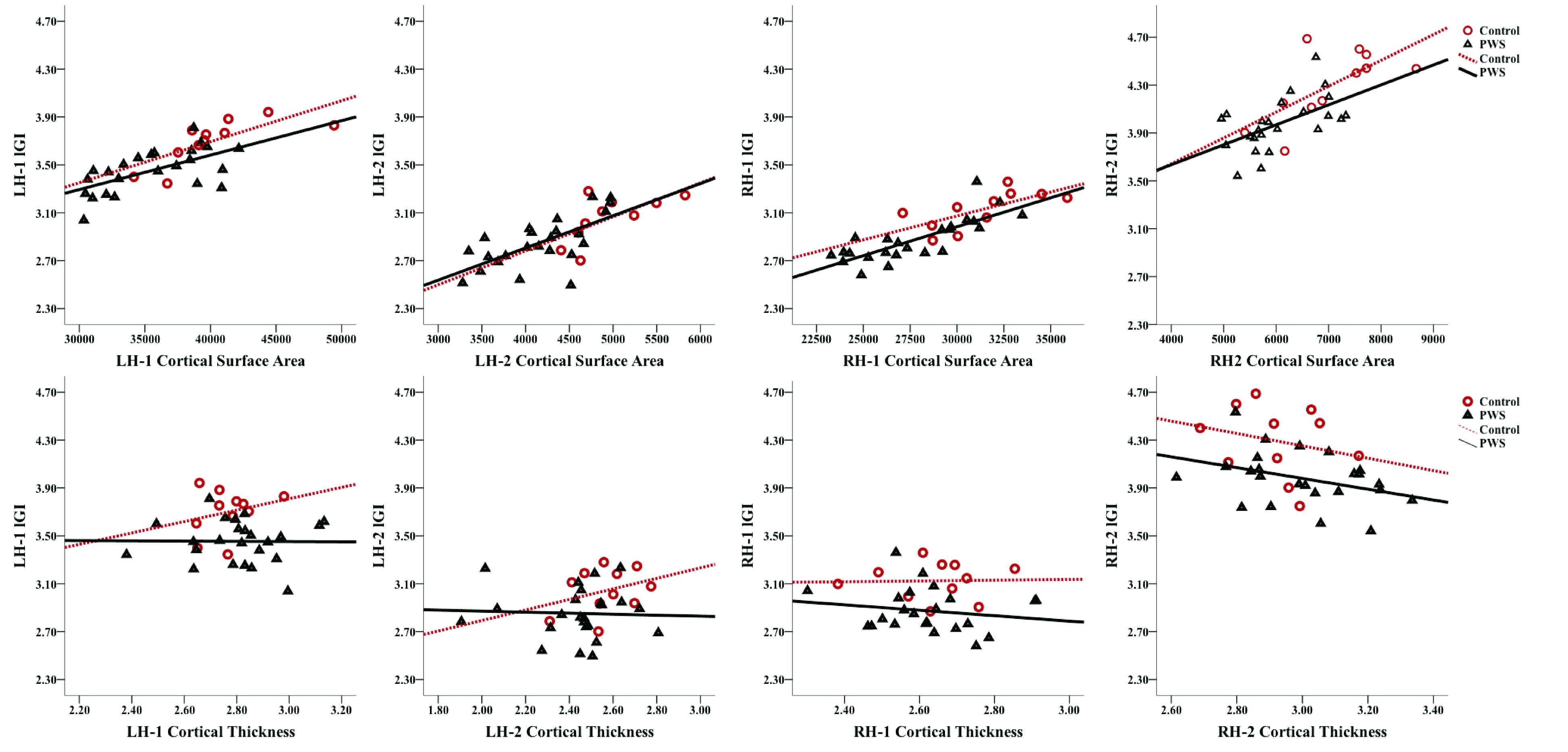

Supplement: Figure S1 — Correlations between lGI, cortical surface area and cortical thickness in children with PWS and healthy controls. LH – Left hemisphere; RH – Right hemisphere. lGI correlated with cortical surface area in PWS and healthy controls, but not with cortical thickness. For corresponding Spearman's rho and p values, together with Fisher's r-to-z transformations for group differences in their relation between lGI and cortical surface area/cortical thickness please see Table 3. (TIF) [file pone.0107320.s001.tif]

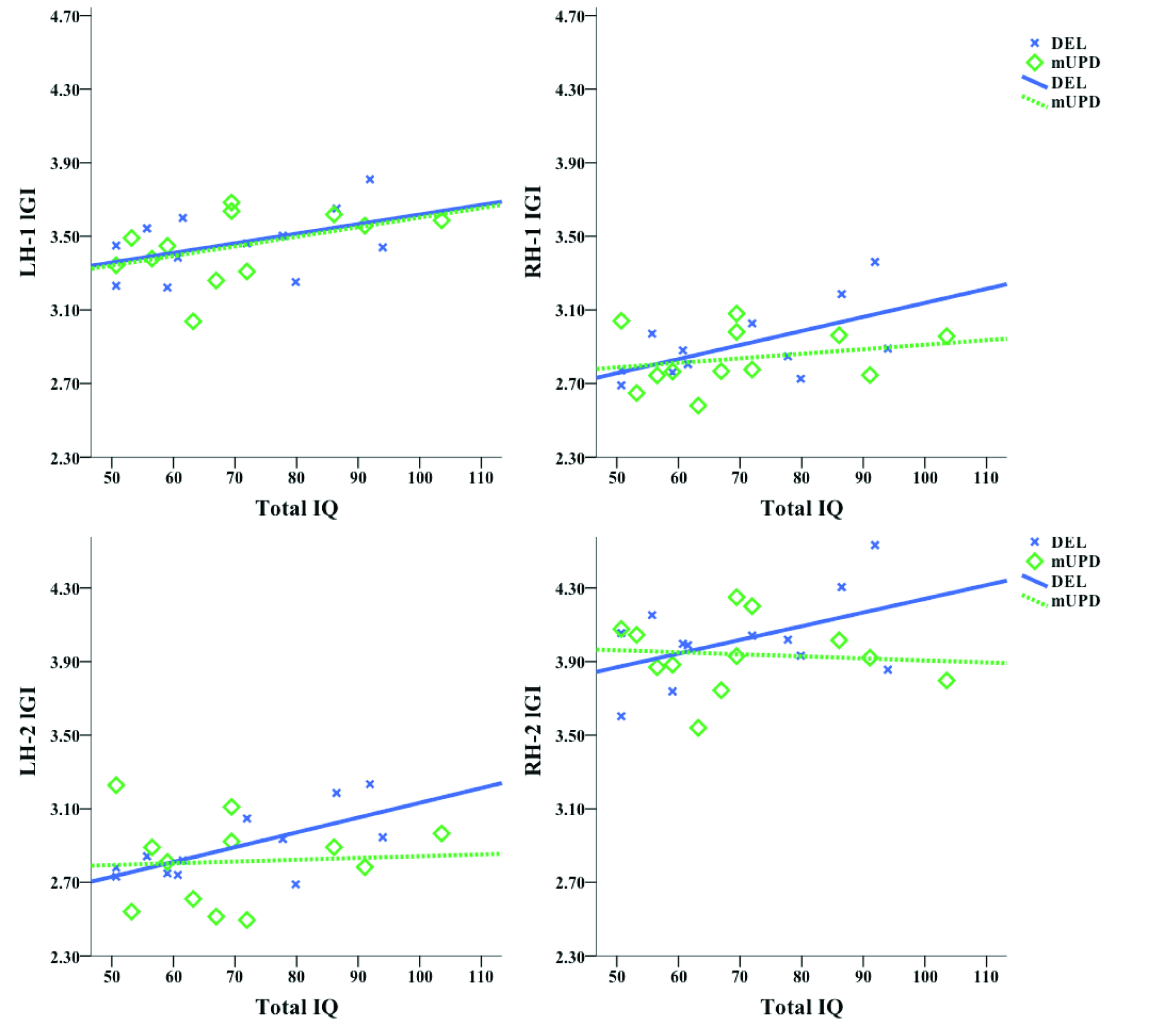

Supplement: Figure S2 — Correlations between lGI and TIQ in children with DEL and mUPD. LH – Left Hemisphere; RH – Right Hemisphere. lGI correlated significantly with Total IQ in children with DEL and mUPD, and no differences were found between the genetic subtypes of PWS. For corresponding Spearman's rho and p values, together with Fisher's r-to-z transformations for subtype differences in their relation between lGI and IQ please see Table S1. (TIF) [file pone.0107320.s002.tif]
