# Supplementary material for: Reduced Cortical Complexity in Children with Prader-Willi Syndrome and Its Association with Cognitive Impairment and Developmental Delay
Source: PLoS One. 2014 Sep 16;9(9):e107320. doi: 10.1371/journal.pone.0107320 (PMC4165760; doi:10.1371/journal.pone.0107320)
Supplement: Table S1 — lGI and IQ relationship within mUPD and DEL groups and group differences. LH – Left hemisphere; RH – Right hemisphere, rho –Spearman's rho. No differences were found between the genetic subtype in the relationship of lGI and IQ measures. (DOCX) [file pone.0107320.s003.docx]

Supplementary Table S1. lGI and IQ relationship within mUPD and DEL groups and group differences.

|  | Total IQ | | | | | | |  |  | Verbal IQ | | | | | | | |  | Performance IQ | | | | | | | |
| --- | --- | --- | --- | --- | --- | --- | --- | --- | --- | --- | --- | --- | --- | --- | --- | --- | --- | --- | --- | --- | --- | --- | --- | --- | --- | --- |
| **lGI cluster** | mUPD | |  | DEL | |  | Fisher’s  r-to z | |  | mUPD | |  | DEL | |  | Fisher’s  r-to z | |  | mUPD | |  | DEL | |  | Fisher’s r-to-z | |
|  | rho | p |  | rho | p |  | z | p |  | rho | p |  | rho | p |  | z | p |  | rho | p |  | rho | p |  | z | p |
| **LH-1** | .44 | .16 |  | .41 | .19 |  | .08 | ns |  | .53 | .08 |  | .45 | .14 |  | .22 | ns |  | .29 | .36 |  | .27 | .39 |  | .05 | ns |
| **LH-2** | .014 | .97 |  | **.59** | **.04** |  | -1.41 | ns |  | .13 | .68 |  | **.68** | **.02** |  | -1.5 | ns |  | .11 | .74 |  | .45 | .15 |  | -.79 | ns |
| **RH-1** | .25 | .43 |  | .55 | .06 |  | -.77 | ns |  | .37 | .23 |  | .52 | .08 |  | -.40 | ns |  | .04 | .90 |  | .48 | .11 |  | -1.02 | ns |
| **RH-2** | -.05 | .89 |  | .22 | .48 |  | -.58 | ns |  | -.03 | .94 |  | .28 | .39 |  | -.67 | ns |  | -.10 | .76 |  | .18 | .58 |  | -.60 | ns |

LH – Left hemisphere; RH – Right hemisphere, rho –Spearman’s rho. No differences were found between the genetic subtype in the relationship of lGI and IQ measures.
