# Supplementary material for: Reduced Cortical Complexity in Children with Prader-Willi Syndrome and Its Association with Cognitive Impairment and Developmental Delay
Source: PLoS One. 2014 Sep 16;9(9):e107320. doi: 10.1371/journal.pone.0107320 (PMC4165760; doi:10.1371/journal.pone.0107320)
Supplement: Table S2 — Correlations between lGI and IQ in clusters with lower lGI in the left hemisphere in patients with PWS. LH – left hemisphere, rho –Spearman's rho. The correlation of lGI and IQ measures (Total, Verbal, Performance) per anatomical area, according to Destrieux anatomical atlas, that clusters comprised of. lGI correlated mainly with Verbal and Total IQ but less with Performance IQ. (DOCX) [file pone.0107320.s004.docx]

Supplementary Table S2. Correlations between lGI and IQ in clusters with lower lGI in the left hemisphere in patients with PWS

|  | **Total IQ** | | **Verbal IQ** | | **Performance IQ** | |
| --- | --- | --- | --- | --- | --- | --- |
| **Area** | **rho** | **p value** | **rho** | **p value** | **rho** | **p value** |
| **LH-1** | **.44** | **.033** | **.50** | **.013** | .30 | .15 |
| Precentral | **.55** | **.006** | **.60** | **.002** | **.41** | **.046** |
| Postcentral | **.49** | **.015** | **.53** | **.008** | .36 | .083 |
| Paracentral | .37 | .075 | **.41** | **.047** | .31 | .15 |
| Supramarginal | .23 | .28 | .29 | .17 | .13 | .54 |
| Superior Parietal | **.53** | **.008** | **.58** | **.003** | .39 | .06 |
| Caudal middle frontal | **.53** | **.007** | **.59** | **.003** | .38 | .07 |
| Rostral middle frontal | **.49** | **.015** | **.53** | **.007** | .32 | .13 |
| Superior Frontal | **.53** | **.008** | **.58** | **.003** | .39 | .06 |
| Precuneus | **.48** | **.020** | **.53** | **.008** | .37 | .08 |
| Posterior Cingulate | .27 | .20 | .32 | .13 | .17 | .44 |
| Caudal anterior cingulate | .23 | .27 | .25 | .24 | .15 | .49 |
| Rostral anterior cingulate | .30 | .16 | .28 | .19 | .24 | .26 |
| Isthmus cingulate | .24 | .25 | .32 | .13 | .11 | .63 |
| Pars opercularis | .39 | .06 | **.43** | **.038** | .27 | .20 |
| Pars triangularis | **.44** | **.033** | **.45** | **.026** | .34 | .10 |
| Insula | .30 | .16 | .38 | .06 | .21 | .33 |
| Superior temporal | -.009 | .97 | .08 | .72 | -.09 | .68 |
| Transverse temporal | .15 | .50 | .25 | .23 | .04 | .86 |
| Middle temporal | -.20 | .34 | -.19 | .38 | -.22 | .30 |
| Medial orbitofrontal | .29 | .18 | .32 | .13 | .17 | .42 |
| Lateral orbitofrontal | .29 | .18 | .38 | .07 | .19 | .37 |
| **LH-2** | .36 | .08 | **.47** | **.022** | .20 | .36 |
| Pericalcarine | .33 | .12 | **.41** | **.048** | .22 | .31 |
| parahippocampal | .04 | .87 | .09 | .69 | -.06 | .80 |
| lingual | .30 | .16 | .40 | .053 | .13 | .54 |
| fusiform | .29 | .17 | **.41** | **.045** | .13 | .56 |

LH – left hemisphere, rho –Spearman’s rho. The correlation of lGI and IQ measures (Total, Verbal, Performance) per anatomical area, according to Destrieux anatomical atlas, that clusters comprised of. lGI correlated mainly with Verbal and Total IQ but less with Performance IQ.
