# Supplementary material for: Reduced Cortical Complexity in Children with Prader-Willi Syndrome and Its Association with Cognitive Impairment and Developmental Delay
Source: PLoS One. 2014 Sep 16;9(9):e107320. doi: 10.1371/journal.pone.0107320 (PMC4165760; doi:10.1371/journal.pone.0107320)
Supplement: Table S3 — Correlations between lGI and IQ in clusters with lower lGI in the right hemisphere in patients with PWS. RH – Right hemisphere, rho –Spearman's rho. The correlation of lGI and IQ measures (Total, Verbal, Performance) per anatomical area, according to Destrieux anatomical atlas, that clusters comprised of. lGI correlated mainly with Verbal and Total IQ, but less with Performance IQ. (DOCX) [file pone.0107320.s005.docx]

Supplementary Table S3. Correlations between lGI and IQ in clusters with lower lGI in the right hemisphere in patients with PWS

|  | **Total IQ** | | **Verbal IQ** | | **Performance IQ** | |
| --- | --- | --- | --- | --- | --- | --- |
| **Area** | **rho** | **p value** | **rho** | **p value** | **rho** | **p value** |
| **RH-1** | .36 | .08 | **.42** | **.044** | .23 | .28 |
| Precentral | **.60** | **.002** | **.61** | **.002** | **.49** | **.016** |
| Postcentral | **.52** | **.009** | **.50** | **.013** | .47 | .022 |
| Paracentral | **.42** | **.039** | **.51** | **.011** | .30 | .16 |
| Caudal middle frontal | **.62** | **.001** | **.70** | **<.001** | **.43** | **.037** |
| Rostral middle frontal | .33 | .12 | .38 | .067 | .13 | .56 |
| Superior frontal | **.47** | **.021** | **.51** | **.010** | .33 | .12 |
| Precuneus | .29 | .16 | .35 | .095 | .21 | .32 |
| Posterior cingulate | .29 | .17 | .35 | .096 | .20 | .35 |
| Caudal anterior cingulate | .13 | .54 | .16 | .45 | .07 | .73 |
| Isthmus cingulate | .16 | .47 | .20 | .36 | .10 | .66 |
| Lingual | .21 | .34 | .24 | .26 | .15 | .48 |
| Parahippocampal | .18 | .40 | .18 | .41 | .20 | .35 |
| Fusiform | .29 | .17 | .32 | .13 | .28 | .19 |
| Entorhinal | .07 | .74 | .12 | .58 | .08 | .71 |
| **RH-2** | .07 | .76 | .11 | .63 | .05 | .82 |
| Inferior parietal | .43 | **.034** | **.48** | **.017** | .30 | .15 |
| Middle temporal | .06 | .79 | .06 | .77 | .03 | .89 |
| Superior temporal | .007 | .98 | .07 | .75 | -.02 | .93 |
| Banks of superior temporal sulcus | .22 | .30 | .22 | .31 | .24 | .26 |

RH – Right hemisphere, rho –Spearman’s rho. The correlation of lGI and IQ measures (Total, Verbal, Performance) per anatomical area, according to Destrieux anatomical atlas, that clusters comprised of. lGI correlated mainly with Verbal and Total IQ, but less with Performance IQ.
